# Early Warning Signs of Schizophrenia: Effective Strategies and the Benefits of Early Treatment

**DOI:** 10.1192/j.eurpsy.2025.2192

**Published:** 2025-08-26

**Authors:** A. H. I. Abu Shehab, A. Baltă, A. B. Ciubară, S. L. Burlea, V. Doina Carina, M. Grigoraș, A. Ciubară

**Affiliations:** 1 Psychiatry, “Elisabeta Doamna” Psychiatry Hospital of Galati; 2 Clinical; 3 orthopaedics and traumatology, Faculty of Medicine and Pharmacy, “Dunărea de Jos” University, Galati; 4 Oral and maxillofacial surgery, 7University of Medicine and Pharmacy “Grigore T. Popa”, Iași; 5 Rheumatology; 6 Psychology; 7 Psychiatry, Faculty of Medicine and Pharmacy, “Dunărea de Jos” University, Galati, Romania

## Abstract

**Introduction:**

Schizophrenia is a severe mental condition marked by a progressive onset of symptoms. Early evaluation and proper management are necessary for improving long-term outcomes and reducing the disorder’s severity. Early detection of prodromal symptoms and the prompt initiation of treatment can substantially influence the evolution of the condition, resulting in improved prognoses and a better quality of life.

**Objectives:**

The paper examines methods for recognizing early indicators of schizophrenia and evaluates the effects of early intervention. The emphasis encompasses comprehending the prevalent prodromal symptoms linked to schizophrenia, assessing diverse early detection techniques, and analysing the advantages of prompt intervention on long-term results.

**Methods:**

A comprehensive examination of existing literature and clinical investigations was performed to identify and delineate prevalent prodromal symptoms of schizophrenia, including social disengagement, cognitive impairments, and atypical thought processes. The assessment examined various early detection instruments, encompassing structured clinician interviews, self-report questionnaires, and neuroimaging methodologies. Furthermore, data from longitudinal studies was examined to ascertain how early intervention may impact the disorder’s course and enhance patient outcomes.

**Results:**

The review realized multiple significant prodromal signs, including social isolation and cognitive impairments. Multiple early detection instruments, including structured interviews and neuroimaging, proved helpful in identifying persons at elevated risk for developing schizophrenia. Timely intervention measures, integrating pharmacological therapies and psychosocial assistance, correlated with a substantial decrease in symptom severity and improved long-term results.

**Conclusions:**

The management of schizophrenia necessitates early identification and intervention. The severity of the disorder and the prognosis can be significantly reduced by recognizing prodromal symptoms and administering effective treatment. In order to enhance recovery and mitigate the effects of schizophrenia on individuals and their families, clinicians should prioritize early detection and early treatment.

**Disclosure of Interest:**

None Declared

